# Evaluating Artificial Intelligence as a First-Pass Reader in Fundus Photograph Screening: A Multireader Workflow Validation Study

**DOI:** 10.3390/diagnostics16172707

**Published:** 2026-08-25

**Authors:** Jihyeon Baek, Richul Oh, Doohyun Park, Kunho Bae

**Affiliations:** 1 VUNO Inc., Seoul 06541, Republic of Korea; jihyeon.baek@vuno.co (J.B.); doohyun.park@vuno.co (D.P.); 2 Department of Ophthalmology, Seoul National University Hospital, 101 Daehak-ro, Jongno-gu, Seoul 03080, Republic of Korea; rcoh91@gmail.com; 3 Mass Eye and Ear and the Department of Ophthalmology, Harvard Medical School, Boston, MA 02114, USA

**Keywords:** artificial intelligence, deep learning, fundus photography, double reading, arbitration, age-related macular degeneration, diabetic retinopathy, retinal vein occlusion, screening

## Abstract

**Background/Objectives:** Double reading with arbitration improves diagnostic reliability in fundus screening but requires repeated human interpretation. Whether artificial intelligence (AI) can serve as a first-pass decision source within this workflow, and whether this applies consistently across retinal diseases with differing inter-reader agreement, remains unclear. **Methods:** In this retrospective study, 6904 color fundus photographs from 2593 patients at a tertiary screening center were analyzed for age-related macular degeneration (AMD), diabetic retinopathy (DR), and retinal vein occlusion (RVO). Three retina specialists independently labeled each image, and an AI system provided binary classifications at a prespecified operating threshold targeting 0.99-sensitivity. In AI–human double reading, the AI and one reader independently interpreted each image, and a second reader arbitrated discordant cases; this was compared with conventional human–human double reading. Three reader combinations were evaluated per disease. **Results:** AI–human reading required 1.02–1.11 human reads per image versus 2.00–2.10 for human–human reading. For DR and RVO, human–human reading yielded sensitivities of 0.974 and 0.982 and specificities of 0.999 and 1.000, respectively. Across AI–human combinations, sensitivity and specificity did not differ significantly from human–human reading (all *p* ≥ 0.05; specificity differences ≤0.001). For AMD (human–human sensitivity 0.845, specificity 1.000), AI–human sensitivity varied: two combinations were higher (0.916 and 0.950; both *p* < 0.001) and one comparable (0.842; *p* = 0.742). AMD specificity remained ≥0.978. **Conclusions:** AI–human reading halved human reading volume without significant loss for DR and RVO; AMD varied by configuration. AI use within double-reading workflows should account for disease-specific inter-reader agreement, reader composition, and operating threshold. This was a single-center, retrospective study, prospective external validation in a multicenter setting is warranted before clinical implementation.

## 1. Introduction

Double reading with arbitration is an established quality-assurance strategy in retinal image interpretation. For example, the English National Health Service Diabetic Eye Screening Programme has institutionalized double reading to maintain diagnostic reliability in population-based retinal screening [1]. This reliability, however, comes at the cost of repeated specialist review: each image generally requires two independent human readings, with additional arbitration when readers disagree.

The need to reduce this burden of repeated reading has motivated interest in whether artificial intelligence (AI) can replace one of the two first-pass human readers while preserving the reliability of double reading [2,3,4]. Although this question has been discussed most prominently in diabetic retinopathy (DR) screening, the same workflow problem applies to other retinal diseases evaluated on color fundus photographs, including age-related macular degeneration (AMD) and retinal vein occlusion (RVO) [5,6,7,8]. For these diseases, however, AI cannot be judged only by standalone diagnostic accuracy; its clinical value depends on whether it can function reliably as part of a multi-reader arbitration workflow [9,10].

Deep learning systems for color fundus photographs have shown strong diagnostic performance for major retinal diseases, but most clinical evaluations have assessed AI either as a standalone diagnostic system or as an aid to an individual clinician [7,11,12,13,14]. Such evaluations do not directly address double reading with arbitration, where diagnostic performance emerges from the interaction among first-pass readers and an arbitrator [10].

A practical implementation of this idea is AI–human double reading with arbitration, in which the AI and one human reader independently classify each image and a second human reader resolves only discordant cases. This design preserves at least one human interpretation for every image while limiting additional human review to cases of AI–human disagreement.

In this study, we applied this framework to AMD, DR, and RVO, three retinal diseases with distinct patterns of inter-reader agreement. We compared AI–human double reading with conventional human–human double reading in terms of diagnostic performance and human reading volume. We also examined whether the workflow’s diagnostic performance and human reading volume varied according to which human readers were paired with the AI, an operational factor that may affect implementation in real-world reading services.

## 2. Materials and Methods

### 2.1. Study Design and Ethical Approval

**Figure 1:**
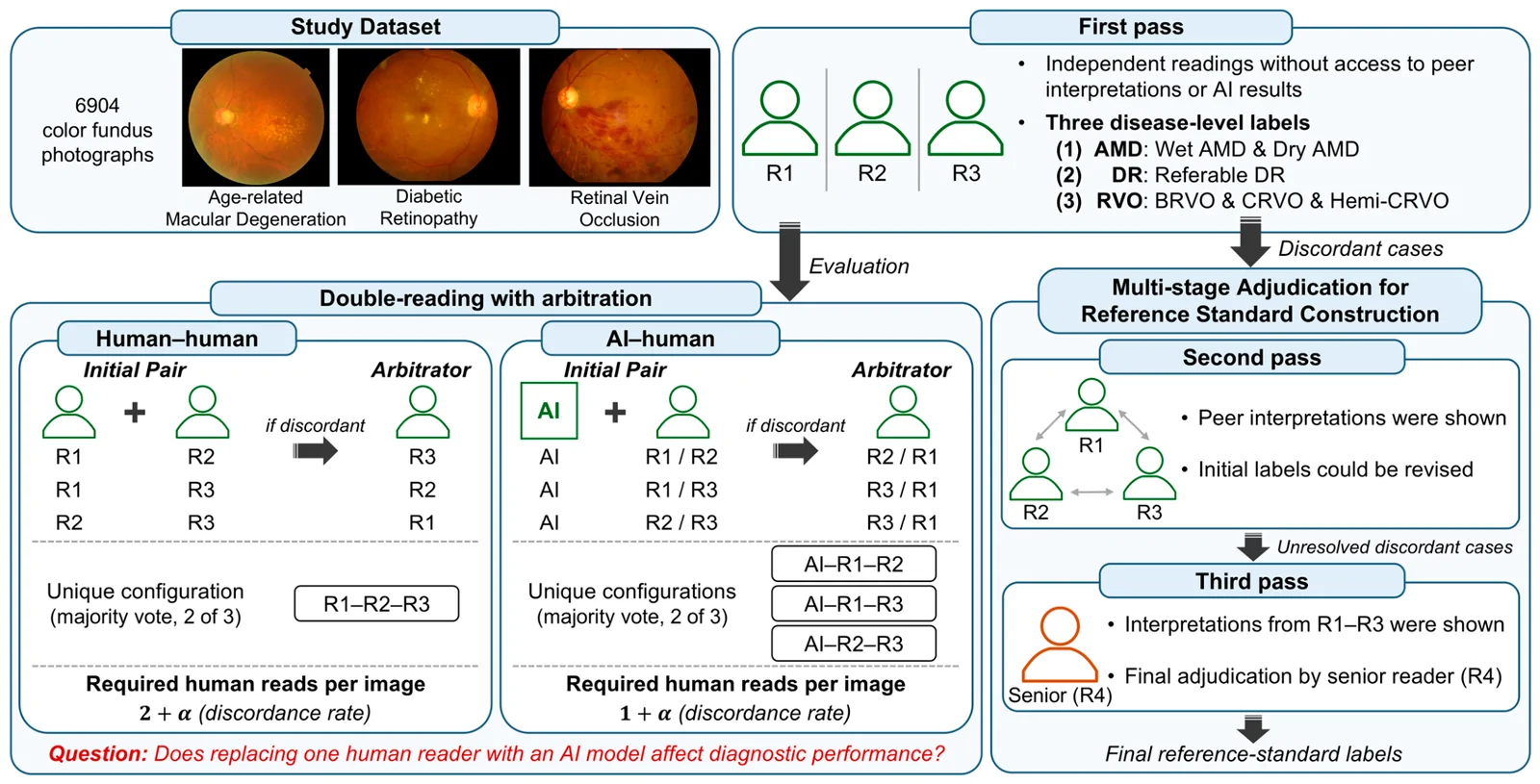
Study design for evaluating a multireader double-reading workflow with and without AI. The reference standard was established by three board-certified retina specialists (Readers 1–3) through a three-step adjudication process, with remaining disagreements resolved by a senior ophthalmologist. Double-reading strategies were reconstructed from the readers’ initial independent labels and the AI output, comparing human–human and AI–human configurations under the same arbitration structure. The hyphenated notation denotes the three decision sources, not a fixed reading order; for example, AI–R1–R2 indicates AI–R1 first-pass reading with R2 arbitration or AI–R2 first-pass reading with R1 arbitration. AI, artificial intelligence; AMD, age-related macular degeneration; DR, diabetic retinopathy; RVO, retinal vein occlusion; BRVO, branch retinal vein occlusion; CRVO, central retinal vein occlusion.

This study was designed to evaluate an AI system as a first-pass reader within a double-reading screening workflow. Double reading, in which two independent first-pass assessments are reconciled by arbitration when they disagree, is a standard quality-assurance approach in image-based screening, and we asked whether one of the two human first-pass readers could be replaced by AI without compromising diagnostic performance. To address this, conventional human–human double reading and AI–human double reading were compared under a common arbitration structure, drawing on the same set of independent reader labels and AI outputs, and their diagnostic accuracy and human reading volume were assessed for AMD, DR, and RVO (Figure 1).

This retrospective study was conducted at Seoul National University Hospital (SNUH) and adhered to the tenets of the Declaration of Helsinki. Institutional Review Board approval was obtained (IRB No. H-2402-005-1506) and the requirement for written informed consent was waived owing to the retrospective nature of the study. Imaging hardware and disease-positive case definitions are reported in Supplementary Methods S1 and S2.

### 2.2. Patient Population and Analysis Sets

A total of 6904 anonymized color fundus photographs from 2593 patients acquired at SNUH between 2004 and 2024 were included. The cohort was independent of all datasets used for AI development, including model training and operating-threshold selection.

Disease-specific analysis sets were defined before workflow performance evaluation. For the AMD analysis, images labeled as geographic atrophy or atrophy-like macular changes were excluded (*n* = 95), because these findings were not included in the predefined AMD target definition evaluated in this study and could not be consistently adjudicated as AMD-related versus non-AMD atrophic or scar-like lesions using the available disease-level labels.

In addition, images were excluded from each disease-specific analysis when a definitive reference-standard label could not be assigned after adjudication. The numbers of indeterminate cases were 131 for AMD, 47 for DR, and 23 for RVO. Indeterminate cases were those for which the senior ophthalmologist was unable to assign a definitive positive or negative adjudicated label, typically because the findings were too borderline to be confidently classified for the target disease. As diagnostic accuracy estimation required a definitive binary reference label, these cases were considered non-evaluable and were excluded from the performance-evaluable analysis set. These exclusions were determined solely by the human adjudication panel according to the reference-standard criteria; the AI system did not participate in identifying or excluding these cases, and the same criteria were applied identically before evaluating the human–human and AI–human workflows.

**Figure 2:**
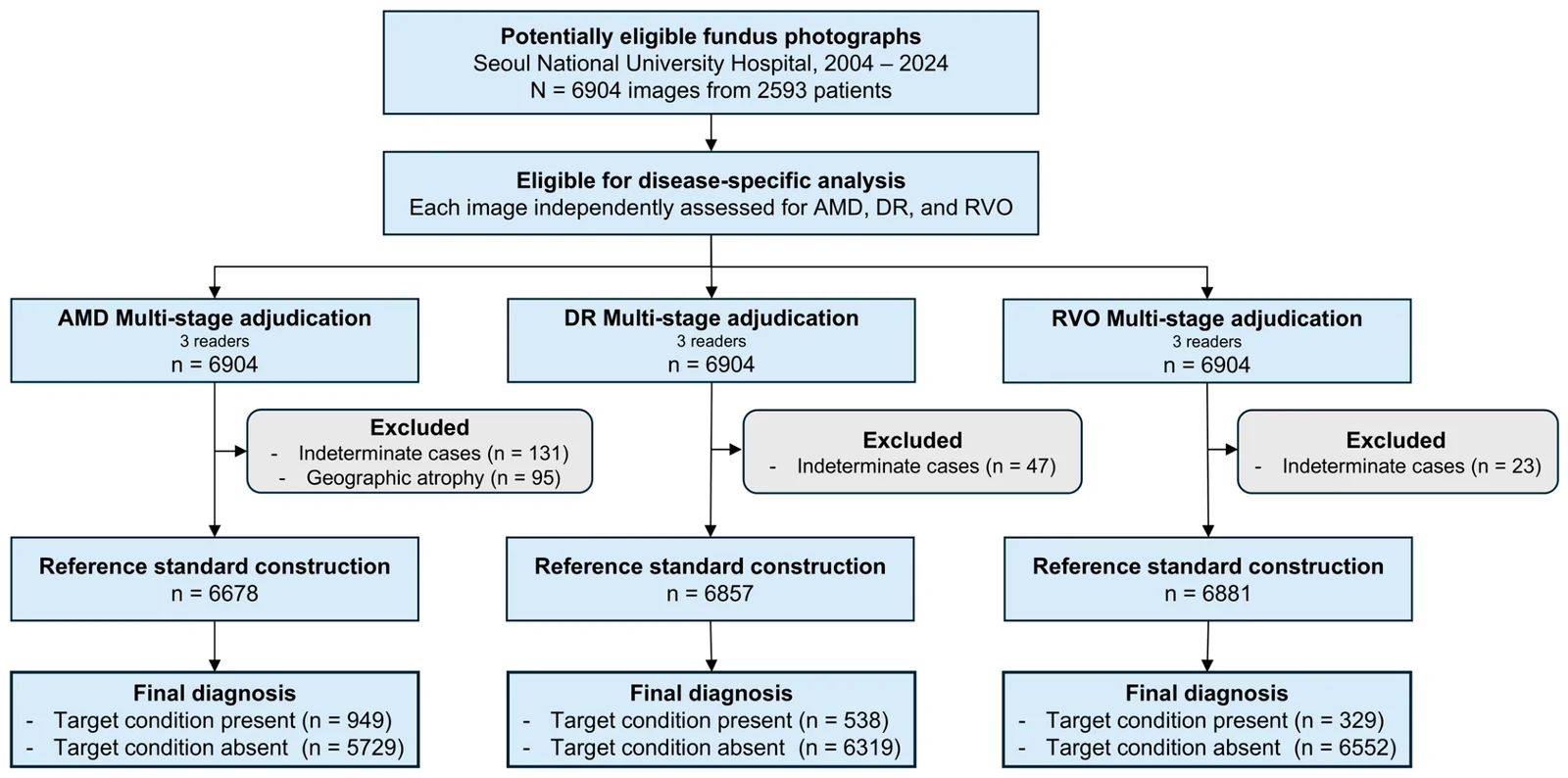
Standards for Reporting of Diagnostic Accuracy (STARD) flow diagram of image selection and disease-specific analysis sets. AMD, age-related macular degeneration; DR, diabetic retinopathy; RVO, retinal vein occlusion.

After these exclusions, the final analyzable samples were 6678 images for AMD (949 positive [14.2%]), 6857 for DR (538 positive [7.8%]), and 6881 for RVO (329 positive [4.8%]) (Figure 2).

### 2.3. AI System and Operating Threshold

The AI system was VUNO Med-FundusDx v1.0.0 (VUNO Inc., Seoul, Republic of Korea), a fundus image analysis software certified by the Ministry of Food and Drug Safety (MFDS), Republic of Korea. For each of AMD, DR, and RVO, binary classifications were generated using prespecified operating thresholds defined before initiation of this study. These thresholds were established independently of the present dataset and were not selected based on the study results.

The primary analysis used prespecified disease-specific operating thresholds corresponding to a target sensitivity of 0.99. These thresholds were selected on an independent validation dataset before this study to prioritize case detection. To assess how threshold choice affected workflow performance, the analyses were repeated using target sensitivities of 0.95 and 0.995. No threshold was selected or recalibrated using the present study cohort, and detailed results are provided in Table S2. A high-sensitivity operating point was prespecified because, in screening-oriented retinal imaging, false-negative errors are generally regarded as more consequential than false-positive errors.

### 2.4. Reference Standard Construction

Each image was independently reviewed by three board-certified retina specialists (Readers 1–3) with 11, 10, and 7 years of subspecialty experience, respectively, without access to AI outputs. A three-step adjudication process established the reference standard. First, all readers independently assigned disease-level labels. Second, discordant cases were re-reviewed after each reader was shown the other readers’ initial assessments. Finally, any remaining disagreements were resolved by a senior ophthalmologist, who assigned the final adjudicated label. These adjudicated labels constituted the reference standard for performance evaluation (Figure 1).

### 2.5. Double-Reading Strategies

Reading strategies were reconstructed using the initial independent labels of Readers 1–3 and the AI output. Three human–human double-reading configurations were evaluated. In each configuration, two readers independently assigned binary labels, and the third reader served as arbitrator when the initial readers disagreed; concordant labels were accepted as the final decision.

AI–human double reading followed the same arbitration framework. In this strategy, the AI system and one human reader served as the two first-pass decision sources. If their binary labels were concordant, the concordant label was accepted as the final workflow decision. If their labels were discordant, arbitration was simulated using the initial label of a second human reader (Figure 1). In both strategies, arbitration was resolved using the arbitrating reader’s own initial independent label, which had been recorded before any adjudication step and without access to the AI output or to the other readers’ decisions, so that both retained an independent human assessment of every image and human adjudication of discordant cases. The AI and human readers therefore functioned as independent decision sources, meaning that each label was generated without sight of any other label and entered the majority rule unchanged, rather than as decision aids, which would have been presented to a reader during interpretation. The term ‘configuration’ denotes only which independently recorded labels were combined under the majority rule and does not imply interaction between the AI and the readers.

Since arbitration in this reconstructed workflow was deferred to the third independent label, the final decision was mathematically equivalent to a majority vote among the three decision sources. Accordingly, configurations that were composed of the same three decision sources produced identical classifications regardless of which human reader was assigned as the first-pass reader and which was assigned as the arbitrator. For example, AI–R1–R2 and AI–R2–R1 yielded the same final diagnostic decision rule because both were based on the same three labels: AI, R1, and R2. Similarly, the three human–human configurations based on R1, R2, and R3 yielded a single unique diagnostic outcome.

However, human reading volume was not necessarily identical across assignments using the same three decision sources. This is because the additional human reader was consulted only when the two first-pass decision sources disagreed. Accordingly, diagnostic performance was summarized at the level of each unique set of decision sources, whereas human reading volume was reported separately according to the assigned first-pass and arbitration roles.

### 2.6. Endpoints and Statistical Analysis

Inter-reader agreement among Readers 1–3 was assessed using Fleiss’ κ and pairwise Cohen’s κ, with 95% confidence intervals (CIs) [15,16]. Sensitivity and specificity of each final diagnostic strategy were calculated against the adjudicated reference standard, with two-sided 95% Wilson score CIs. Human reading volume was calculated as the number of mandatory first-pass human reads plus one additional human read when the first-pass decision sources were discordant. Mean human reads per image therefore equaled 2 + α for human–human double reading and 1 + α for AI–human double reading, where α denotes the observed first-pass discordance rate. (Figure 1; Section 2.5).

For each disease, AI–human final diagnostic decisions were compared with human–human final diagnostic decisions using paired McNemar tests on the same images [17]. McNemar tests for sensitivity and specificity were performed separately among reference-positive and reference-negative images, respectively. AI–human discordant cases were additionally examined by summarizing standalone AI errors and their correction within the arbitration workflow (Table S3) and by qualitatively reviewing representative cases together with attention-based saliency maps of the AI predictions (Figure S1). Clinical net benefit was additionally evaluated using decision curve analysis [18,19], computing the net benefit of each final double-reading strategy across a range of threshold probabilities (Figure S2). As a post hoc analysis, non-inferiority of each AI–human strategy relative to human–human double reading was assessed separately for sensitivity and specificity as the paired difference in proportions, with exact 95% CIs obtained from the Clopper–Pearson interval for the proportion of discordant pairs favoring the AI–human strategy, rescaled to the difference [20]. The non-inferiority margins, prespecified in regulated trials of autonomous retinal-imaging AI [21], were −0.05 for sensitivity and −0.025 for specificity. The margin was not prespecified in the present study, and the analysis complements rather than replaces the paired McNemar tests.

This study was a secondary analysis of previously collected data; therefore, no a priori sample size calculation was performed. The sample size was determined by the number of eligible cases available in the source cohort. Analyses were performed at the image level, which was the unit of the reconstructed screening workflow. Patient- and eye-level linkage identifiers were not retained in the de-identified analytic dataset. All statistical analyses were performed in R version 4.5.3.

## 3. Results

### 3.1. Per-Reader Positive Call Rate and Inter-Reader Agreement

Per-reader positive call rates for each disease are summarized in Table 1, with individual reader values provided in Table S1. For AMD, positive call rates varied from 7.8% to 17.4%, compared with an adjudicated reference-positive proportion of 14.2%, indicating greater reader-dependent variability in AMD. In contrast, positive call rates were closely clustered and aligned with the adjudicated reference-positive proportions for DR (7.0–7.6% vs. 7.8%) and RVO (4.6–4.7% vs. 4.8%).

This disease-specific pattern was reflected in the inter-reader agreement statistics. Fleiss’ κ was 0.658 (95% CI, 0.644–0.672) for AMD, 0.896 (0.882–0.910) for DR, and 0.971 (0.957–0.984) for RVO, indicating substantial agreement for AMD and almost perfect agreement for DR and RVO according to standard interpretive criteria [22]. Pairwise Cohen’s κ values across the three reader pairs were consistent with this pattern, ranging from 0.541 to 0.744 for AMD, 0.876 to 0.934 for DR, and 0.966 to 0.979 for RVO.

### 3.2. Human Reading Volume of Double-Reading Strategies

By design, reconstructed human–human double reading required two initial human reads for every image, whereas AI–human double reading required one initial human read for every image. In both strategies, an additional human read was required only when the two first-pass decision sources were discordant and arbitration was triggered. First-pass discordance (α), which determines arbitration volume, occurred in 5.7–10.3% of images for human–human reader pairs and in 6.8–11.2% for AI–human pairs in AMD, in 0.9–1.7% and 1.8–2.3% respectively in DR, and in 0.2–0.3% and 7.3% respectively in RVO. Accordingly, human–human double reading required 2.00 to 2.10 human reads per image, whereas AI–human double reading required 1.02 to 1.11, corresponding to a 46% to 49% reduction in human reading events (Table 2). Although AI–human discordance was higher for RVO, total human reading volume remained lower because only one mandatory human first-pass read was required.

### 3.3. Diagnostic Performance of Double-Reading Strategies

The final diagnostic classification was identical for configurations composed of the same three decision sources, irrespective of first-pass versus arbitration role assignment. Therefore, diagnostic performance was summarized according to unique decision-source combinations. For each disease, this included one human–human comparator based on R1, R2, and R3 and three AI–human combinations based on AI–R1–R2, AI–R1–R3, and AI–R2–R3 (Table 2).

For AMD, human–human double reading achieved a sensitivity of 0.845 (95% CI, 0.821–0.867) and a specificity of 1.000 against the adjudicated reference standard. Among the three AI–human combinations, sensitivity varied according to the human readers included in the decision-source set. Two AI–human combinations showed significantly higher sensitivity than human–human double reading, with sensitivities of 0.916 and 0.950, respectively (both *p* < 0.001). The highest sensitivity was observed for AI–R1–R3, reaching 0.950 (95% CI, 0.935–0.963). The remaining AI–human combination showed a sensitivity of 0.842, which was not significantly different from human–human double reading (*p* = 0.742). Specificity was lower for all three AI–human combinations than for human–human double reading, although it remained 0.978 or higher.

For DR, all three AI–human combinations showed sensitivity estimates ranging from 0.959 to 0.968, compared with 0.974 for human–human double reading. AI–human and human–human reading did not differ significantly in sensitivity or specificity. Specificity remained 0.999 or higher across all AI–human combinations, with absolute specificity differences of 0.001 or less compared with human–human double reading.

For RVO, all three AI–human combinations showed sensitivity estimates ranging from 0.985 to 0.994, compared with 0.982 for human–human double reading. AI–human and human–human reading did not differ significantly in sensitivity. Specificity was 1.000 for all AI–human combinations, identical to that of human–human double reading.

### 3.4. Secondary and Sensitivity Analyses

Across the alternative AI operating points summarized in Table S2, the effect of threshold choice was disease-dependent. For AMD, raising the AI target sensitivity from 0.95 to 0.995 increased workflow sensitivity from 0.854 to 0.940 for AI–R1–R2 and from 0.921 to 0.973 for AI–R1–R3, while workflow specificity fell from 0.992 to 0.967 and from 0.991 to 0.966 respectively, and mean human reads per image rose from 1.04–1.07 to 1.10–1.17. For DR, the same change increased workflow sensitivity by 0.027 to 0.044, and for RVO by 0.003 to 0.006, while mean human reads per image remained between 1.02 and 1.08.

**Figure 3:**
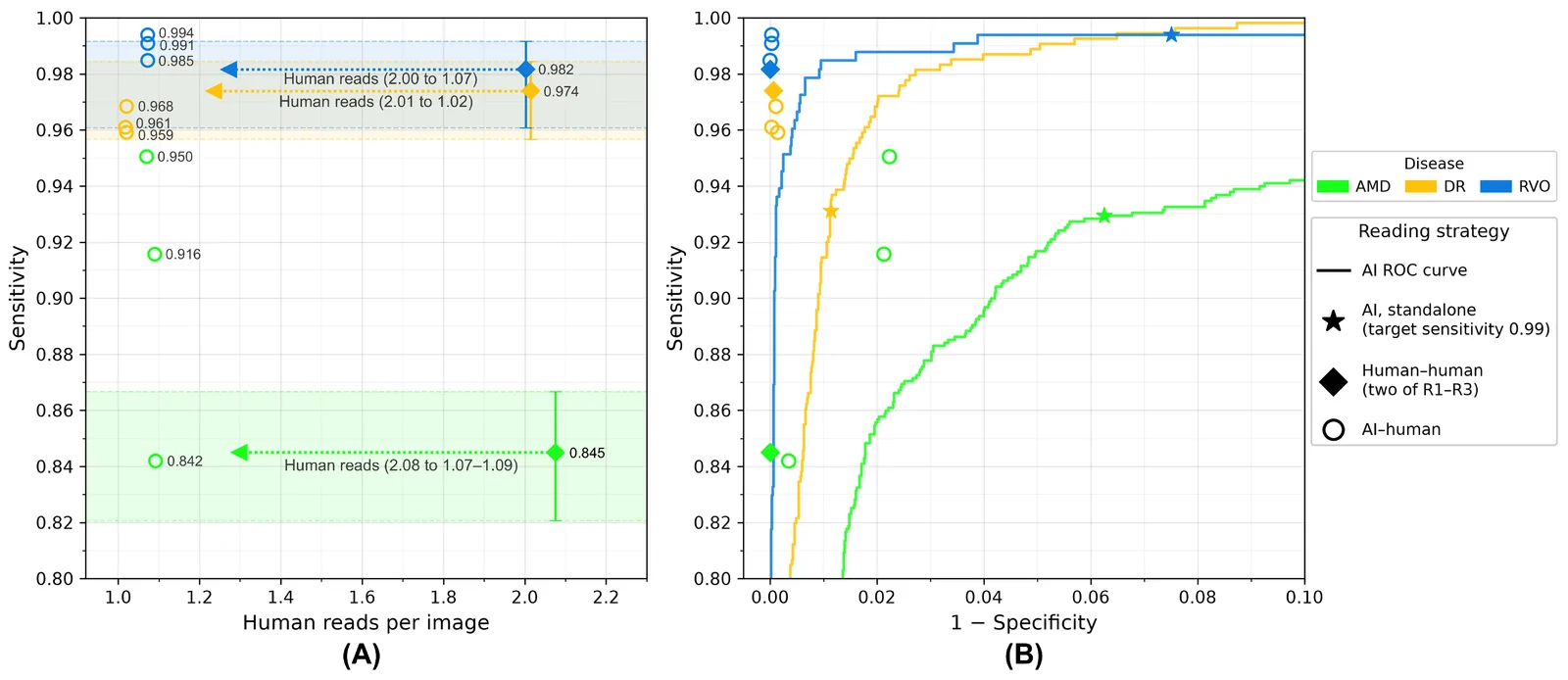
Comparison of human–human and AI–human double-reading strategies. (**A**) Diagnostic sensitivity according to the number of human reads per image. Diamonds indicate human–human double reading using two of the three human readers, and circles indicate AI–human double reading using AI plus one human reader. (**B**) Receiver operating characteristic curves of the AI model for each disease, with markers indicating the operating points of the human–human and AI–human double-reading strategies. AMD, age-related macular degeneration; DR, diabetic retinopathy; RVO, retinal vein occlusion; AI, artificial intelligence.

With the AI output evaluated alone, before arbitration, at the primary operating point, AI sensitivity and specificity were 0.929 and 0.938 for AMD, 0.931 and 0.989 for DR, and 0.994 and 0.925 for RVO (Table S1; Figure 3B). Most standalone AI false positives were corrected within the arbitration workflow: 230–338 of 358 (64–94%) for AMD, 65–70 of 72 (90–97%) for DR, and 490–492 of 492 (99.6–100%) for RVO across the three reader configurations. Correction of standalone AI false negatives was less consistent (AMD, 2–20 of 67 [3–30%]; DR, 17–20 of 37 [46–54%]; RVO, 0–1 of 2) (Table S3). Representative discordant cases, together with attention-based saliency maps of the AI predictions, are shown in Figure S1.

Decision curve analysis showed that, across the clinically relevant range of threshold probabilities, both double-reading strategies yielded net benefit well above the default strategies of referring all or no images. For DR and RVO, the net benefit of AI–human double reading was essentially equivalent to that of human–human double reading (absolute difference ≤ 0.001 in net benefit), whereas for AMD, AI–human double reading provided higher net benefit at lower threshold probabilities (Figure S2).

In the post hoc analysis, all AI–human configurations were non-inferior to human–human double reading for sensitivity at the −0.05 margin and for specificity at the −0.025 margin (Table S4). CIs were defined for 17 of the 18 comparisons. In the remaining comparison, RVO specificity for AI–R2–R3, the two strategies classified every reference-negative image identically, giving an exact paired difference of zero.

## 4. Discussion

The present study demonstrates that AI–human double reading with arbitration can substantially reduce human reading burden compared with conventional human–human double reading, while maintaining comparable diagnostic performance in selected settings. Standalone AI performance does not capture how AI functions within a multi-reader workflow [9,23]; we therefore evaluated AI–human double reading against an adjudicated reference standard while simultaneously quantifying human reading burden, allowing assessment of both diagnostic performance and operational efficiency. The key implication is not that AI should replace clinical oversight, but rather that it can function as an independent first-pass reader within a structured arbitration workflow [24]. As a result, repeated specialist review is reduced while human oversight is preserved.

The value of this workflow is particularly relevant in settings where double reading is routinely required, such as research cohorts, reading centers, and clinical-trial grading [1,25]. Unlike routine clinical practice, these environments prioritize highly reproducible reference labels and therefore depend heavily on multiple independent readers [10,25]. In such contexts, incorporating AI as an independent first-pass decision source may offer a favorable balance between reliability and efficiency, preserving human oversight and the benefits of double reading while substantially reducing specialist workload.

The observed trade-off varied across diseases. For DR and RVO, where inter-reader agreement was relatively high, AI–human double reading closely replicated the diagnostic behavior of human–human double reading while requiring fewer human reads. In these conditions, the main contribution of AI appeared to be operational, reducing repeated specialist review without a statistically significant loss of sensitivity or specificity in the evaluated configurations. AMD showed a more configuration-dependent pattern. Interpretation of AMD on color fundus photographs is often influenced by borderline findings and reader-specific decision thresholds, resulting in greater inter-reader variability [25,26]. Consequently, workflow performance depended more strongly on the specific set of decision sources combined with the AI, suggesting that reader composition may be particularly important in diseases characterized by lower agreement among human readers. This configuration dependence appeared to be driven largely by differences in reader-specific positive-call thresholds rather than by random variation alone. Among the three readers, AMD positive-call rates were 17.4% for R1, 7.8% for R2, and 12.5% for R3 (Table S1). The two combinations that significantly exceeded human–human sensitivity (AI–R1–R2 and AI–R1–R3) shared one reader in common, whereas the combination that did not improve sensitivity (AI–R2–R3) excluded that reader. This pattern suggests that when the high-sensitivity AI was combined with more conservative readers, its additional true-positive detections were more often overturned at arbitration, limiting the net sensitivity gain; differences in the severity distribution of borderline AMD findings across the reader-defined case sets may also have contributed.

The high-sensitivity AI threshold was selected to reduce missed cases [3,4,24,27,28], although this lowered the specificity of the AI when used alone. In the double-reading workflow, most AI false positives were corrected by human review, allowing workflow specificity to remain high. Although standalone AI specificity was as low as 0.925 for RVO and 0.938 for AMD, workflow specificity remained 0.978 or higher across all diseases. Together with the approximately 50% reduction in human reading volume, these findings suggest that high-sensitivity AI may provide an efficient first-pass decision source in selected double-reading workflows. Accordingly, the near-perfect workflow specificity should be interpreted as a property of the arbitration workflow rather than of the AI system alone, which operated at a deliberately high-sensitivity, lower-specificity point.

A related consideration is that, as a standalone classifier, the AI system did not reach its prespecified operating target of 0.99 sensitivity for two of the three diseases, achieving a standalone sensitivity of 0.929 for AMD and 0.931 for DR, although it reached 0.994 for RVO. Two factors likely contributed to this gap. First, the operating threshold was fixed during development, and domain shift between the development data and the present single-center cohort—including differences in patient population and imaging devices—can lower sensitivity at a fixed threshold [10]. Second, the reference standard was constructed through strict adjudication by three retina specialists, producing stringent disease labels against which standalone sensitivity is conservatively estimated [10,19]. This shortfall did not, however, carry over to the reconstructed workflow, where the AI served as only one of two first-pass decision sources and discordant cases were referred to an additional human reader who could compensate for its false negatives. Consequently, workflow sensitivity was maintained or improved relative to human–human double reading, reaching 0.950 for the best-performing AMD configuration (AI–R1–R3) and remaining comparable for DR and RVO.

These findings suggest that AI systems intended for double-reading workflows should be developed and evaluated not only for standalone diagnostic performance, but also for their complementarity with human readers and their performance after arbitration. Because arbitration can correct AI errors only when they are not shared by the human readers, future development should consider disease-specific patterns of agreement and disagreement between AI and readers. Reader-level analyses further suggest that AI operating thresholds may need to be considered in relation to reader configuration. In the present study, retina specialists generally demonstrated high specificity but more variable sensitivity, indicating that individual readers differed in their decision thresholds for borderline or subtle findings. A high-sensitivity AI may provide complementary information when paired with readers who have lower positive call rates, whereas more specific AI thresholds may be preferable in configurations where false-positive classifications are a greater concern. Although prospective optimization of reader-specific threshold selection was beyond the scope of this study, the findings suggest that AI operating thresholds should be viewed as a configurable component of workflow design rather than a fixed technical parameter [23,29].

The adjudicated reference standard also warrants consideration. The initial labels of the participating readers contributed to reference-standard construction, whereas AI outputs did not; this design may have favored the human–human comparator. Nevertheless, AI–human double reading achieved diagnostic performance estimates that were similar to the human–human comparator for DR and RVO, and showed higher sensitivity in two of the three AMD configurations, albeit with lower specificity.

An important methodological feature of this study is that AI and human readers operated independently. AI outputs were not displayed to the human readers at any stage and therefore could not influence their initial judgments. The observed differences across configurations resulted from combining different sets of independently recorded labels rather than from changes in reader behavior. The workflow thus evaluated AI as an independent decision source within a double-reading framework, rather than as a decision-support tool presented to clinicians during interpretation. This distinction is important because exposure to AI recommendations may influence clinician judgment and introduce automation bias [30,31,32]. By maintaining independence between AI and human interpretations, the present design minimized this source of bias and allowed a clearer assessment of the AI system’s contribution to the reconstructed collective decision-making process. In a prospective implementation in which the arbitrating reader is shown the preceding AI and human decisions, both would function as decision aids, and the potential influence of automation bias would need to be evaluated. Such bias can be limited by preserving the sequence evaluated here. Each reader would record and confirm an independent interpretation before any AI output or other reader’s label is disclosed, with disclosure restricted to the adjudication of discordant cases. Where post-disclosure revision is permitted, recording the reader’s pre- and post-disclosure labels would allow the direction and accuracy of any change to be monitored.

The sequencing and presentation of AI outputs remain important considerations for real-world implementation. In practice, AI-first, human-first, and fully independent parallel-reading workflows may differ in efficiency, reader behavior, and susceptibility to automation bias [32,33,34]. The arbitration-based approach evaluated in this study most closely reflects an independent parallel-reading structure, in which AI and human judgments are generated separately and discordant cases are resolved by additional human review. Therefore, the optimal implementation strategy is likely to depend on institutional priorities, including reading efficiency, error reduction, arbitration capacity, and tolerance for false-positive referrals.

Clinical implementation would require more than validation of diagnostic performance. Readers and arbitrators would need training on disease-specific AI error patterns, threshold-related false-positive classifications, and the management of discordant or ungradable images. Regulatory review would also be needed to determine whether use of the AI system as one of two first-pass decision sources is consistent with its authorized intended use in each jurisdiction. Services would need to adapt staffing and arbitration pathways because replacing one mandatory first-pass human read reduces routine reading volume but may increase or redistribute arbitration demand, as observed for RVO. Technical deployment would require integration with image acquisition and reporting systems, predefined routing of ungradable images to human review, and governance for software, model, and threshold updates. Because such updates may alter workflow performance, local validation would be required before implementation.

The generalizability of these findings will depend on the setting in which the workflow is implemented. Differences in disease prevalence and referral criteria may affect arbitration volume, referral burden, and net benefit across tertiary hospitals, community clinics, and primary care screening programs. Differences in patient characteristics, disease spectrum, imaging devices, and image acquisition protocols may also affect AI performance through domain shift. In addition, other AI algorithms may have different operating thresholds, calibration, and error patterns. The diagnostic performance and reading volume observed in this study should therefore not be directly extrapolated beyond the evaluated AI system, cohort, devices, and reader panel. Nevertheless, the broader workflow concept of replacing one first-pass human reader with AI while retaining human arbitration may be relevant to other settings. Prospective validation will be required for each AI algorithm, population, device environment, and clinical workflow.

Within each implementation setting, the AI operating threshold should be selected according to the relative importance of case detection, false-positive referrals, and available arbitration capacity. A lower sensitivity target may reduce false-positive classifications and the need for human review, which may be preferable in high-volume screening programs with limited specialist capacity. A higher sensitivity target prioritizes case detection but may reduce specificity and increase arbitration volume. This trade-off was most apparent for AMD, where inter-reader agreement was lower, and was less pronounced for DR and RVO. Higher sensitivity thresholds may therefore be appropriate in reading centers, clinical trial grading, or other settings in which avoiding missed cases and achieving reproducible final classifications take priority over reading burden. The prespecified target sensitivity of 0.99 should thus be regarded as a balance between these competing priorities rather than an optimal threshold for all applications.

This study has several limitations. First, the workflow was evaluated retrospectively using reconstructed reading strategies rather than real-time reader interactions. Although this design minimized automation bias, it may not fully reflect clinical implementation or the behavioral changes that could occur when AI outputs are incorporated into routine practice. Accordingly, the evaluated workflow should be regarded as a test framework rather than a proposed implementation model; actual adoption would require local adaptation based on clinical needs, available resources, regulatory and organizational context, and input from relevant stakeholders. The reported efficiency measure represents the number of human reading events rather than interpretation, reporting, or turnaround time, which were not recorded. Prospective reader studies are therefore needed to evaluate these time-based measures, referral burden, and clinical safety under real-world conditions. Patient-level outcomes also require prospective follow-up to determine whether additional case detection results in earlier treatment or improved visual outcomes. Although reduced human reading volume may translate into lower specialist time and costs, this cannot be determined from the present data. Formal economic evaluation will require reading-time and staffing costs, downstream referral costs and yields, and patient-level outcomes.

Second, patient- and eye-level linkage identifiers were not available in the de-identified analytic dataset and could not be reconstructed; therefore, repeated images from the same patient or eye could not be identified, and within-patient correlation could not be modeled using clustered approaches such as generalized estimating equations or mixed-effects models. Given the relatively small average number of images per patient, the impact of this limitation may be limited, but it cannot be excluded. Third, formal probabilistic calibration of the AI confidence scores (e.g., calibration curves, Brier score, and expected calibration error) was not assessed, because the workflow was evaluated at fixed, prespecified operating thresholds.

Fourth, the adjudicated reference standard incorporated labels from the participating human readers, whereas AI outputs were not. This potential incorporation bias may have favored the human–human comparator and should be addressed in future studies using an independent or blinded reference standard. Fifth, excluding indeterminate images without a definitive reference-standard label means the specificity estimated here may be marginally optimistic relative to an unselected screening population that also includes ambiguous cases. Sixth, images with geographic atrophy or atrophy-like macular changes were excluded from the AMD analysis (Section 2.2; Supplementary Methods S2), so the AMD results do not extend to atrophic late AMD.

Seventh, the evaluation was limited to the three retinal diseases for which the evaluated AI system provides predictions (AMD, DR, and RVO); applicability to comprehensive ophthalmic screening therefore remains unknown and would require models covering additional targets. Eighth, the results apply specifically to the evaluated commercial AI system and its prespecified operating thresholds; comparable performance with other deep-learning architectures cannot be assumed without separate validation. Lastly, the images were acquired over a 20-year period (2004–2024) at a single tertiary referral center using multiple imaging devices and acquisition protocols, which may have introduced temporal or device-related domain shift; prospective multicenter validation across multiple institutions, ethnically diverse populations, imaging devices, and screening settings will therefore be required before clinical implementation.

## 5. Conclusions

AI–human double reading with arbitration substantially reduced repeated human reading while preserving diagnostic reliability for DR and RVO and demonstrating reader-configuration-dependent performance for AMD. These findings suggest that AI may be best implemented as an independent, structurally embedded decision source within double-reading workflows, rather than as an isolated standalone diagnostic system. Its clinical value will depend on the disease target, reader configuration, the selected AI operating threshold, and prospective multicenter validation within the intended screening or reading environment.

## Figures and Tables

**Table 1:** Study population, positive call-rate range among Readers 1–3, and inter-reader agreement.

Disease	N (Reference Positive, %)	Positive Call-Rate Range Among Readers 1–3	Fleiss’ κ R1–R2–R3 (95% CI)	Cohen’s κ R1–R2 (95% CI)	Cohen’s κ R1–R3 (95% CI)	Cohen’s κ R2–R3 (95% CI)
AMD	6678 (949, 14.2%)	7.8–17.4%	0.658 (0.644–0.672)	0.541 (0.512–0.570)	0.744 (0.721–0.766)	0.691 (0.662–0.720)
DR	6857 (538, 7.8%)	7.0–7.6%	0.896 (0.882–0.910)	0.934 (0.918–0.951)	0.876 (0.853–0.898)	0.877 (0.855–0.900)
RVO	6881 (329, 4.8%)	4.6–4.7%	0.971 (0.957–0.984)	0.967 (0.953–0.982)	0.966 (0.951–0.980)	0.979 (0.967–0.990)

CI, confidence interval; AMD, age-related macular degeneration; DR, diabetic retinopathy; RVO, retinal vein occlusion. The per-reader positive call rate is the proportion of images included in the disease-specific analysis for which an individual reader independently judged the target disease to be present according to the prespecified criteria. A positive call was defined as early or intermediate AMD according to the Beckman classification or neovascular AMD, moderate nonproliferative DR or worse and/or diabetic macular edema, or fundus findings compatible with branch or central retinal vein occlusion. Geographic atrophy cases were excluded from the AMD analysis. Full definitions are provided in Supplementary Methods S2.

**Table 2:** Diagnostic performance and human reading volume of reconstructed double-reading strategies against the adjudicated reference standard.

Disease	Double-Reading Strategy	Arbitrator on Disagreement	Human Reads per Image	Sensitivity (95% CI)	*p* ^†^	Specificity (95% CI)	*p* ^†^
AMD	Human–human	R1–R2–R3	2.06–2.10	0.845 (0.821–0.867)	-	1.000 (0.999–1.000)	-
	AI–human	AI–R1–R2	1.07–1.11	**0.916** **(0.896–0.932)**	**<0.001**	0.979 (0.975–0.982)	<0.001
		AI–R1–R3	1.07–1.07	**0.950** **(0.935–0.963)**	**<0.001**	0.978 (0.973–0.981)	<0.001
		AI–R2–R3	1.07–1.11	0.842 (0.817–0.864)	0.742	0.997 (0.995–0.998)	<0.001
DR	Human–human	R1–R2–R3	2.01–2.02	0.974 (0.957–0.984)	-	0.999 (0.998–1.000)	-
	AI–human	AI–R1–R2	1.02–1.02	0.961 (0.941–0.974)	0.065	1.000 (0.999–1.000)	0.500
		AI–R1–R3	1.02–1.02	0.968 (0.950–0.980)	0.607	0.999 (0.998–0.999)	0.453
		AI–R2–R3	1.02–1.02	0.959 (0.939–0.973)	0.077	0.999 (0.997–0.999)	0.062
RVO	Human–human	R1–R2–R3	2.00–2.00	0.982 (0.961–0.992)	-	1.000 (0.999–1.000)	-
	AI–human	AI–R1–R2	1.07–1.07	0.991 (0.974–0.997)	0.250	1.000 (0.999–1.000)	0.500
		AI–R1–R3	1.07–1.07	0.994 (0.978–0.998)	0.219	1.000 (0.999–1.000)	0.500
		AI–R2–R3	1.07–1.07	0.985 (0.965–0.993)	1.000	1.000 (0.999–1.000)	1.000

R1, R2, and R3 denote the three board-certified retina specialists, respectively. The hyphenated notation denotes the three decision sources, not a fixed reading order; for example, AI–R1–R2 indicates AI–R1 first-pass reading with R2 arbitration or AI–R2 first-pass reading with R1 arbitration. Diagnostic performance is invariant to the order, whereas human reads per image vary and are reported as a range. Sensitivity and specificity are reported with Wilson score 95% confidence intervals (CIs). ^†^ *p* values are two-sided paired McNemar tests on the same images comparing each AI–human double-reading strategy with the human–human double-reading strategy. These tests are based on paired comparisons, whereas the 95% CIs are estimated separately for each strategy; the CIs should therefore not be directly compared with the corresponding *p* values. Values shown in bold indicate statistically significant improvements (*p* < 0.05) of the AI–human over the human–human double-reading strategy. For RVO, a specificity of 1.000 corresponds to 0 false positives among the 6552 reference-negative images for the human–human and AI–R2–R3 strategies, and 2 for AI–R1–R2 and AI–R1–R3 (specificity 0.99969, rounded to 1.000).

## Data Availability

The original color fundus photographs are not publicly available due to institutional data protection policies and patient privacy regulations. De-identified tabular data, including the comprehensive reading results used for statistical analyses, are available from the corresponding author on reasonable request.

## Supplementary Materials

The following supporting information can be downloaded at https://www.mdpi.com/article/10.3390/diagnostics16172707/s1, Supplementary Methods S1: Imaging hardware; Supplementary Methods S2: Disease-positive case definitions; Table S1: Diagnostic performance of individual readers and artificial intelligence (AI) systems with target sensitivity of 0.99; Table S2: Diagnostic performance of AI–human double-reading strategies across AI operating thresholds. Table S3: Standalone artificial intelligence (AI) errors and their correction within the AI–human double-reading arbitration workflow. Figure S1: Qualitative error analysis of representative AI–human discordant cases. Figure S2: Decision curve analysis of net benefit for the double-reading strategies. Table S4. Post hoc non-inferiority analysis of artificial intelligence (AI)–human versus human–human double reading. References [35,36,37,38] are cited in the supplementary materials.

## Abbreviations

The following abbreviations are used in this manuscript:

AI	Artificial intelligence
AMD	Age-related macular degeneration
CI	Confidence interval
DR	Diabetic retinopathy
RVO	Retinal vein occlusion
