# Supplementary material for: Evaluating Artificial Intelligence as a First-Pass Reader in Fundus Photograph Screening: A Multireader Workflow Validation Study
Source: Diagnostics (Basel). 2026 Aug 25;16(17):2707. doi: 10.3390/diagnostics16172707 (PMC13564521; doi:10.3390/diagnostics16172707)
Supplement: Supplementary file 1 [file diagnostics-16-02707-s001.zip › diagnostics-4441360-supplementary.pdf]

Supplementary Methods S1. Imaging hardware

Color fundus photographs (CFPs) were acquired using multiple cameras at Seoul National University Hospital, including the VISUCAM 524 (Carl Zeiss Meditec AG, Jena, Germany), TRC-50DX and TRC-50DX Type IA (Topcon Corporation, Tokyo, Japan), and VX-10α and nonmyd 7 (Kowa Company, Ltd., Nagoya, Japan), under mixed mydriatic and nonmydriatic conditions. Images judged ungradable due to poor focus, severe media opacity, illumination artifact, or off-center fixation were excluded from the analysis.

Supplementary Methods S2. Disease-positive case definitions

Age-related macular degeneration (AMD). AMD-positive was defined as fundus findings consistent with early or intermediate AMD per the Beckman Initiative classification, or with neovascular (exudative) late AMD (medium or large drusen, retinal pigment epithelial changes, or neovascular AMD). Geographic atrophy was not included in the AMD-positive definition [1]. Geographic atrophy in this cohort was etiologically heterogeneous, encompassing typical AMD-related geographic atrophy and myopic patchy atrophy and chorioretinal (laser) scars that could not be reliably distinguished at the disease level. For this reason, cases positive for geographic atrophy were excluded from the AMD analysis rather than classified as positive or negative. Eyes without these findings were classified as AMD-negative.

Diabetic retinopathy (DR). DR-positive was defined as moderate nonproliferative diabetic retinopathy (NPDR) or worse and/or diabetic macular edema (DME), corresponding to referral-warranted disease in the International Clinical Diabetic Retinopathy severity scale [2]. CFPs graded as normal, mild NPDR, or non-DR retinal abnormalities were classified as DR-negative.

Retinal vein occlusion (RVO). RVO-positive was defined by fundus findings compatible with

central retinal vein occlusion (CRVO) or branch retinal vein occlusion (BRVO), including dilated tortuous veins with sectoral or panretinal hemorrhage, cotton-wool spots, and disc edema in the affected territory [3,4]. Eyes without these findings were classified as RVO-negative.

Supplementary Table S1. Diagnostic performance of individual readers and artificial intelligence (AI) systems with target sensitivity of 0.99.

Disease	Rater	Positive Call Rate (%)	Sensitivity	Specificity	Accuracy
Age-related macular degeneration (AMD)	Reader 1	17.4	0.967	0.958	0.959
	Reader 2	7.8	0.532	0.997	0.931
	Reader 3	12.5	0.854	0.996	0.975
	AI	18.6	0.929	0.938	0.936
Diabetic retinopathy (DR)	Reader 1	7.6	0.950	0.998	0.994
	Reader 2	7.3	0.920	0.999	0.993
	Reader 3	7.0	0.877	0.998	0.989
	AI	8.4	0.931	0.989	0.984
Retinal vein occlusion (RVO)	Reader 1	4.7	0.973	1.000	0.998
	Reader 2	4.6	0.964	1.000	0.998
	Reader 3	4.6	0.973	1.000	0.999
	AI	11.9	0.994	0.925	0.928

Values are for the primary AI operating point (target sensitivity 0.99) and are computed against the adjudicated reference standard on the disease-specific analysis sets (AMD, n = 6,678; DR, n = 6,857; RVO, n = 6,881). Readers 1–3 are the three board-certified retina specialists, each reading independently and without access to AI outputs. The per-reader positive call rate is the proportion of images included in the disease-specific analysis for which an individual reader independently judged the target disease to be present according to the prespecified criteria. A positive call was defined as early or intermediate AMD according to the Beckman classification or neovascular AMD, moderate nonproliferative DR or worse and/or diabetic macular edema, or fundus findings compatible with branch or central retinal vein occlusion. Geographic atrophy cases were excluded from the AMD analysis. Full definitions are provided in Supplementary Methods S2. AI, artificial intelligence; AMD, age-related macular degeneration; DR, diabetic retinopathy; RVO, retinal vein occlusion.

Supplementary Table S2. Diagnostic performance of artificial intelligence (AI)–human double-reading strategies across AI operating thresholds.

Double-reading strategy		AI target sensitivity	AMD: Human Reads per image	AMD: Sensitivity (95% CI)	AMD: $P^{\dagger}$	AMD: Specificity (95% CI)	AMD: $P^{\dagger}$	DR: Human Reads per image	DR: Sensitivity (95% CI)	DR: $P^{\dagger}$	DR: Specificity (95% CI)	DR: $P^{\dagger}$	RVO: Human Reads per image	RVO: Sensitivity (95% CI)	RVO: $P^{\dagger}$	RVO: Specificity (95% CI)	RVO: $P^{\dagger}$
			**Age-related macular degeneration (AMD)**					**Diabetic retinopathy (DR)**					**Retinal vein occlusion (RVO)**				
Human–human	R1–R2–R3	-	2.06–2.10	0.845 (0.821–0.867)	-	1.000 (0.999–1.000)	-	2.01–2.02	0.974 (0.957–0.984)	-	0.999 (0.998–1.000)	-	2.00–2.00	0.982 (0.961–0.992)	-	1.000 (0.999–1.000)	-
AI–human	AI–R1–R2	0.950	1.06–1.07	0.854 (0.830–0.875)	0.526	0.992 (0.989–0.994)	**< 0.001**	1.02–1.02	0.941 (0.917–0.958)	**< 0.001**	1.000 (0.999–1.000)	0.500	1.02–1.02	0.988 (0.969–0.995)	0.625	1.000 (0.999–1.000)	1.000
		0.990	1.07–1.11	0.916 (0.896–0.932)	**< 0.001**	0.979 (0.975–0.982)	**< 0.001**	1.02–1.02	0.961 (0.941–0.974)	0.065	1.000 (0.999–1.000)	0.500	1.07–1.07	0.991 (0.974–0.997)	0.250	1.000 (0.999–1.000)	0.500
		0.995	1.10–1.17	0.940 (0.923–0.953)	**< 0.001**	0.967 (0.962–0.971)	**< 0.001**	1.02–1.02	0.968 (0.950–0.980)	0.508	0.999 (0.998–1.000)	1.000	1.08–1.08	0.991 (0.974–0.997)	0.250	1.000 (0.999–1.000)	0.500
	AI–R1–R3	0.950	1.04–1.06	0.921 (0.902–0.936)	**< 0.001**	0.991 (0.988–0.993)	**< 0.001**	1.02–1.02	0.939 (0.915–0.956)	**< 0.001**	1.000 (0.999–1.000)	1.000	1.02–1.02	0.991 (0.974–0.997)	0.453	1.000 (0.999–1.000)	1.000
		0.990	1.07–1.07	0.950 (0.935–0.963)	**< 0.001**	0.978 (0.973–0.981)	**< 0.001**	1.02–1.02	0.968 (0.950–0.980)	0.607	0.999 (0.998–0.999)	0.453	1.07–1.07	0.994 (0.978–0.998)	0.219	1.000 (0.999–1.000)	0.500
		0.995	1.10–1.12	0.973 (0.960–0.981)	**< 0.001**	0.966 (0.961–0.970)	**< 0.001**	1.02–1.03	0.981 (0.966–0.990)	0.424	0.998 (0.997–0.999)	0.065	1.08–1.08	0.994 (0.978–0.998)	0.219	1.000 (0.999–1.000)	0.500
	AI–R2–R3	0.950	1.04–1.07	0.797 (0.770–0.821)	**< 0.001**	0.998 (0.997–0.999)	**< 0.001**	1.02–1.02	0.928 (0.902–0.947)	**< 0.001**	0.999 (0.998–1.000)	1.000	1.02–1.02	0.979 (0.957–0.990)	1.000	1.000 (0.999–1.000)	1.000
		0.990	1.07–1.11	0.842 (0.817–0.864)	0.742	0.997 (0.995–0.998)	**< 0.001**	1.02–1.02	0.959 (0.939–0.973)	0.077	0.999 (0.997–0.999)	0.062	1.07–1.07	0.985 (0.965–0.993)	1.000	1.000 (0.999–1.000)	1.000
		0.995	1.12–1.17	0.852 (0.828–0.874)	0.296	0.995 (0.993–0.996)	**< 0.001**	1.02–1.02	0.972 (0.955–0.983)	1.000	0.998 (0.997–0.999)	**0.031**	1.08–1.08	0.985 (0.965–0.993)	1.000	1.000 (0.999–1.000)	1.000

R1, R2, and R3 denote the three board-certified retina specialists, respectively. The hyphenated notation and the role-assignment convention are as defined in Table 2 of the main text: diagnostic performance is invariant to the order of the three decision sources, whereas human reads per image vary and are reported as a range. Sensitivity and specificity are reported with Wilson score 95% confidence intervals (CIs). $^{\dagger}P$ values are two-sided paired McNemar tests on the same images comparing each AI–human double reading strategy with the human–human double reading strategy. These tests are based on paired comparisons whereas the 95% confidence intervals are estimated separately for each strategy, so the confidence intervals should not be directly compared with the corresponding P values. Bold type indicates $P < 0.05$.

Supplementary Table S3. Standalone artificial intelligence (AI) errors and their correction within the AI–human double-reading workflow with arbitration.

Disease	Standalone AI false positives (n)	AI false positives corrected by arbitration, n (%)	Standalone AI false negatives (n)	AI false negatives corrected by arbitration, n (%)
AMD	358	230–338 (64–94)	67	2–20 (3–30)
DR	72	65–70 (90–97)	37	17–20 (46–54)
RVO	492	490–492 (99.6–100)	2	0–1 (0–50)

Ranges span the three AI–human reader configurations (AI–R1–R2, AI–R1–R3, AI–R2–R3). An AI error was counted as corrected when the final arbitrated majority-vote workflow decision matched the adjudicated reference standard; percentages are relative to the corresponding number of standalone AI errors. AI, artificial intelligence; AMD, age-related macular degeneration; DR, diabetic retinopathy; RVO, retinal vein occlusion.

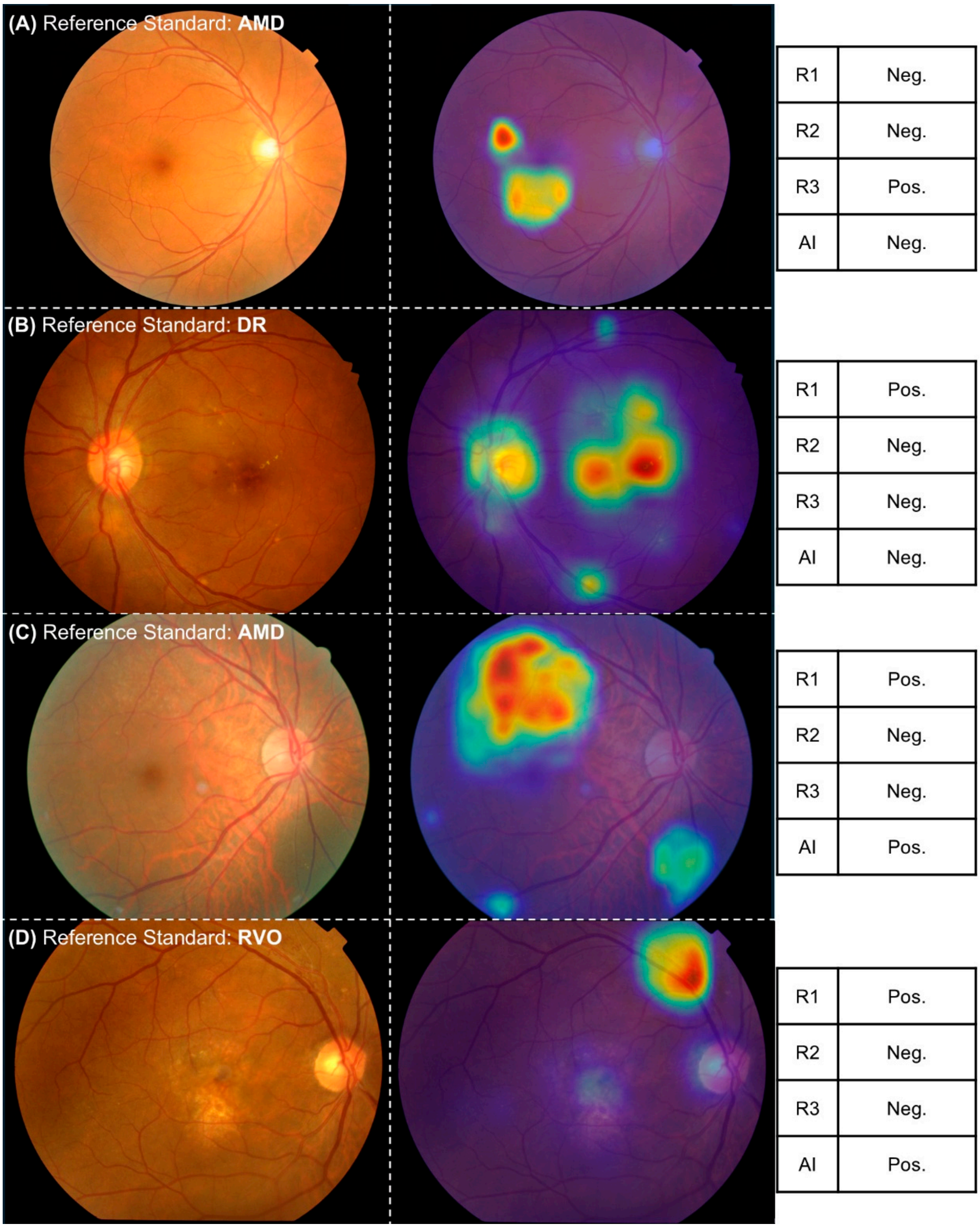

Supplementary Figure S1. Qualitative analysis of representative artificial intelligence (AI)–human discordant cases, with attention-based saliency maps of the AI prediction. Pos., reference standard positive; Neg., reference standard negative; AMD, age-related macular degeneration; DR, diabetic retinopathy; RVO, retinal vein occlusion.

For each case, the original color fundus photograph (left) is shown alongside the corresponding

attention-based saliency map (right), in which warmer colors denote the retinal regions that contributed most strongly to the AI's disease prediction. The table within each panel reports the independent calls of the three retina specialists (R1, R2, R3), the AI's classification and the adjudicated reference standard. Relative to the reference standard, the standalone AI classification was a false negative in (A) AMD and (B) DR and a true positive in (C) AMD and (D) RVO, with the saliency map localizing to the causative lesion in the latter two. The final double-reading decision depended on which independently recorded reader labels were combined with the AI output. Some label combinations resulted in an incorrect majority decision, whereas others resulted in the correct decision, indicating that workflow performance reflected the combination of independent labels rather than any influence of AI on the readers. Image-level correction rates across the reader configurations are quantified in Supplementary Table S3.

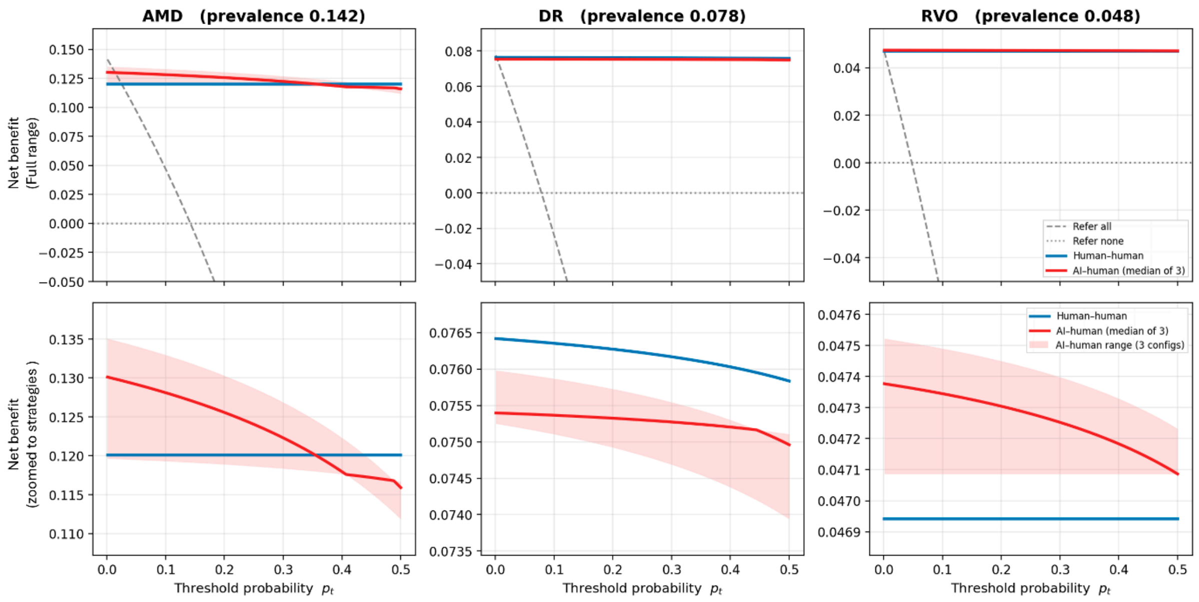

Supplementary Figure S2. Decision curve analysis of the net benefit of human–human and artificial intelligence (AI)–human double-reading strategies for each disease. AMD, age-related macular degeneration; DR, diabetic retinopathy; RVO, retinal vein occlusion.

The threshold probability p_t is the minimum probability of disease at which referral is judged worthwhile and reflects the relative harm of an unnecessary referral versus a missed case (harm ratio = $p_t/(1-p_t)$). Net benefit is the true-positive proportion minus the false-positive proportion weighted by $p_t/(1-p_t)$, expressed per patient on the scale of true positives; zero corresponds to the "Refer none" strategy and negative values indicate net harm. For each disease, net benefit is plotted against p_t for human–human double reading (majority vote of Readers 1–3) and AI–human double reading (majority vote of AI and two readers; the line and shaded band show the median and range across the three reader configurations AI–R1–R2, AI–R1–R3, and AI–R2–R3, with AI at the primary 0.99 target-sensitivity operating point). The top row shows the full range, in which both strategies lie well above the "Refer all" and "Refer none" default strategies; the bottom row rescales the y-axis to the strategy curves to resolve their difference. Because each strategy yields a single

binary decision, its net benefit is fully determined by its sensitivity, specificity, and disease prevalence (Table 2), and the curves therefore express those operating characteristics in net-benefit units weighted by threshold probability. The two strategies are compared in Section 3.4 of the main text across the clinically relevant range of p_t.

Supplementary Table S4. Post hoc non-inferiority analysis of artificial intelligence (AI)–human versus human–human double reading.

Disease	Strategy	Metric	Paired difference (95% CI)	Non-inferior†
AMD	AI–R1–R2	Sensitivity	+0.0706 (+0.0517 to +0.0853)	Yes
AMD	AI–R1–R2	Specificity	−0.0213 (−0.0213 to −0.0200)	Yes
AMD	AI–R1–R3	Sensitivity	+0.1054 (+0.0947 to +0.1091)	Yes
AMD	AI–R1–R3	Specificity	−0.0223 (−0.0223 to −0.0211)	Yes
AMD	AI–R2–R3	Sensitivity	−0.0032 (−0.0160 to +0.0102)	Yes
AMD	AI–R2–R3	Specificity	−0.0035 (−0.0035 to −0.0023)	Yes
DR	AI–R1–R2	Sensitivity	−0.0130 (−0.0195 to +0.0007)	Yes
DR	AI–R1–R2	Specificity	+0.0003 (−0.0002 to +0.0003)	Yes
DR	AI–R1–R3	Sensitivity	−0.0056 (−0.0188 to +0.0099)	Yes
DR	AI–R1–R3	Specificity	−0.0005 (−0.0010 to +0.0005)	Yes
DR	AI–R2–R3	Sensitivity	−0.0149 (−0.0254 to +0.0014)	Yes
DR	AI–R2–R3	Specificity	−0.0008 (−0.0008 to +0.0000)	Yes
RVO	AI–R1–R2	Sensitivity	+0.0091 (−0.0038 to +0.0091)	Yes
RVO	AI–R1–R2	Specificity	−0.0003 (−0.0003 to +0.0002)	Yes
RVO	AI–R1–R3	Sensitivity	+0.0122 (−0.0052 to +0.0181)	Yes
RVO	AI–R1–R3	Specificity	−0.0003 (−0.0003 to +0.0002)	Yes
RVO	AI–R2–R3	Sensitivity	+0.0030 (−0.0074 to +0.0090)	Yes
RVO	AI–R2–R3	Specificity	+0.0000 (no discordant pairs)	Yes

Values are paired differences in the indicated metric (AI–human minus human–human double reading), computed among reference-positive images for sensitivity and reference-negative images for specificity, with 95% confidence intervals obtained by inverting the same exact conditional binomial test used for the McNemar comparisons in Table 2 of the main text: an exact Clopper–Pearson interval for the proportion of discordant pairs favoring the AI–human strategy, rescaled to the difference. Each interval excludes zero when the corresponding McNemar test reaches $P < 0.05$. Being conditional on the number of discordant pairs, these intervals are narrower than unconditional intervals and skewed about the point estimate, which coincides with a confidence bound where one strategy accounts for all discordant pairs. For RVO specificity with AI–R2–R3 the two strategies classified every reference-negative image identically, so there were no discordant pairs and no interval is defined. †Non-inferiority was concluded when the lower confidence bound exceeded the margin prespecified in regulated trials of autonomous retinal-imaging AI: −0.05 for sensitivity and −0.025 for specificity. These margins were not prespecified in the present study, so this analysis is exploratory. R1, R2, and R3 denote the three board-certified retina specialists. CI, confidence interval; AMD, age-related macular degeneration; DR, diabetic retinopathy; RVO, retinal vein occlusion.

References

1. Ferris, F.L.; Wilkinson, C.P.; Bird, A.; Chakravarthy, U.; Chew, E.; Csaky, K.; Sadda, S.R. Clinical Classification of Age-Related Macular Degeneration. *Ophthalmology* **2013**, *120*, 844–851, doi:10.1016/j.ophtha.2012.10.036.

2. Wilkinson, C.P.; Ferris, F.L.; Klein, R.E.; Lee, P.P.; Agardh, C.D.; Davis, M.; Dills, D.; Kampik, A.; Pararajasegaram, R.; Verdaguer, J.T. Proposed International Clinical Diabetic Retinopathy and Diabetic Macular Edema Disease Severity Scales. *Ophthalmology* **2003**, *110*, 1677–1682, doi:10.1016/S0161-6420(03)00475-5.

3. Rogers, S.L.; McIntosh, R.L.; Lim, L.; Mitchell, P.; Cheung, N.; Kowalski, J.W.; Nguyen, H.P.; Wang, J.J.; Wong, T.Y. Natural History of Branch Retinal Vein Occlusion: An Evidence-Based Systematic Review. *Ophthalmology* **2010**, *117*, 1094-1101.e5, doi:10.1016/j.ophtha.2010.01.058.

4. McIntosh, R.L.; Rogers, S.L.; Lim, L.; Cheung, N.; Wang, J.J.; Mitchell, P.; Kowalski, J.W.; Nguyen, H.P.; Wong, T.Y. Natural History of Central Retinal Vein Occlusion: An Evidence-Based Systematic Review. *Ophthalmology* **2010**, *117*, 1113-1123.e15, doi:10.1016/j.ophtha.2010.01.060.
